# Association Between F‐SIRI and Adverse Prognosis in Patients With Chronic Heart Failure

**DOI:** 10.1002/clc.70166

**Published:** 2025-06-16

**Authors:** Xiaoli Liu, Heyu Chu, Guo Song, Yi Wang, Junfeng Duan, Xi Tan, Xue Bao, Biao Xu, Rong Gu

**Affiliations:** ^1^ Department of Cardiology, Nanjing Drum Tower Hospital, the Affiliated Hospital of Medical School Nanjing University Nanjing China; ^2^ Department of Cardiology Nanjing Drum Tower Hospital Clinical College of Nanjing Medical University Nanjing China; ^3^ Department of Cardiology Nanjing Drum Tower Hospital Clinical College of Nanjing University of Chinese Medicine Nanjing China; ^4^ Department of General practice Shenzhen Hospital of Southern Medical University Shenzhen China

**Keywords:** all‐cause death, cardiovascular death, chronic heart failure, fibrinogen, major adverse cardiac and cerebral events, systematic inflammation response index

## Abstract

**Aims:**

High plasma fibrinogen and systemic inflammation response index (F‐SIRI) has been proposed as a novel prognostic factor in resectable gastric cancer. However, available data on the prognostic value of F‐SIRI in chronic heart failure (CHF) patients is limited. We aimed to conduct a retrospective cohort study exploring the correlation between F‐SIRI and prognosis in CHF individuals.

**Methods:**

We consecutively enrolled 1589 hospitalized patients (aged 66 ± 8 years, 32.9% women) with CHF from January 1, 2019 to August 31, 2022 in this single‐center retrospective study. SIRI was calculated with the formula (monocyte count*neutrophil count/lymphocyte count). The primary endpoints encompassed all‐cause death, the major adverse cardiac and cerebral events (MACCEs) and cardiovascular death. The association between F‐SIRI and the risk of developing adverse outcomes were explored using four multivariate‐adjusted Cox proportional hazard models.

**Results:**

During a median follow‐up of 687 days, 207 all‐cause deaths, 462 MACCEs and 136 cardiovascular deaths were recorded. After adjusting for potential confounding factors, only risk of all‐cause death remained significantly associated with higher levels of F‐SIRI. The hazard ratios (HRs) for the highest F‐SIRI group (F‐SIRI = 2) versus the lowest F‐SIRI group (F‐SIRI = 0) were 2.37 (95% confidence interval [CI], 1.46−3.83; *p* < 0.001) for all‐cause death. The addition of F‐SIRI could increase the prognostic ability for all‐cause death on the basis of traditional risk factors.

**Conclusions:**

F‐SIRI is a significant predictor of all‐cause death but has limited predictive value for MACCEs and cardiovascular death in CHF patients.

## Graphical abstract

F‐SIRI, which combines fibrinogen and systematic inflammation response index, is a significant predictor of all‐cause death but has limited predictive value for MACCEs and cardiovascular death in patients With chronic heart failure.

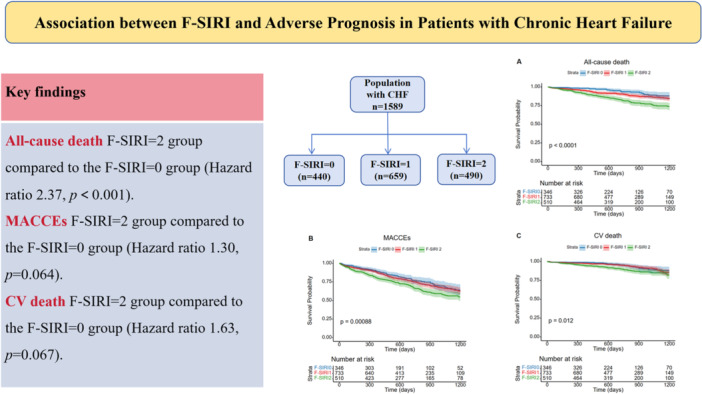

## 1 Introduction

Heart failure (HF) imposes an increasingly heavy financial burden on both individuals and society. As a leading cause of morbidity and mortality, HF affected 64.3 million people in 2017 worldwide [1]. Therefore, more accurate risk stratification strategies should be developed to achieve individualized treatment, which is vital to improve the prognosis of a large population of patients diagnosed with HF.

The presence of systemic inflammation is intricately linked to HF. Over the past few decades, a multitude of pro‐inflammatory cytokines were found to be elevated in individuals diagnosed with HF, including CRP, IL‐1, IL‐6, galectin 3, and so forth, which further confirmed the important role of systemic low‐grade inflammation in the development and progression of HF [2, 3, 4, 5, 6]. Numerous clinical trials investigating the potential therapeutic impact of anti‐inflammatory treatments in HF have been conducted so far and some of them have achieved encouraging results, such as the CANTOS trial [7]. Several indices, comprising the systemic immune‐inflammatory index (SII), neutrophil‐to‐lymphocyte ratio (NLR), lymphocyte‐to‐monocyte ratio (LMR), and others, have been proposed to clinically assess the degree of inflammation in patients with HF. These indices are based on inflammatory cell count and platelet count [8, 9, 10]. Among these indicators, systemic inflammation response index (SIRI) has emerged as an independent factor in predicting overall mortality and intensive care unit hospitalization time for elderly individuals (>60 years old) with chronic HF (CHF) [11]. However, the prognostic significance of SIRI in whole‐age CHF patients remains uncertain.

Previous research has indicated that fibrinogen (FIB) could potentially serve as an cardiovascular risk predictor among individuals diagnosed with coronary artery disease (CAD) [12, 13, 14]. The inflammation index F‐SIRI, composed of fibrinogen and SIRI, demonstrates a favorable prognostic value in individuals with gastric cancer [15]. Considering there is a scarcity of clinical research examining the role of F‐SIRI in HF patients, we conducted this retrospective study to explore the correlation between F‐SIRI and prognosis in individuals with CHF.

## 2 Materials and Methods

### 2.1 Study Design and Population

**Figure 1:**
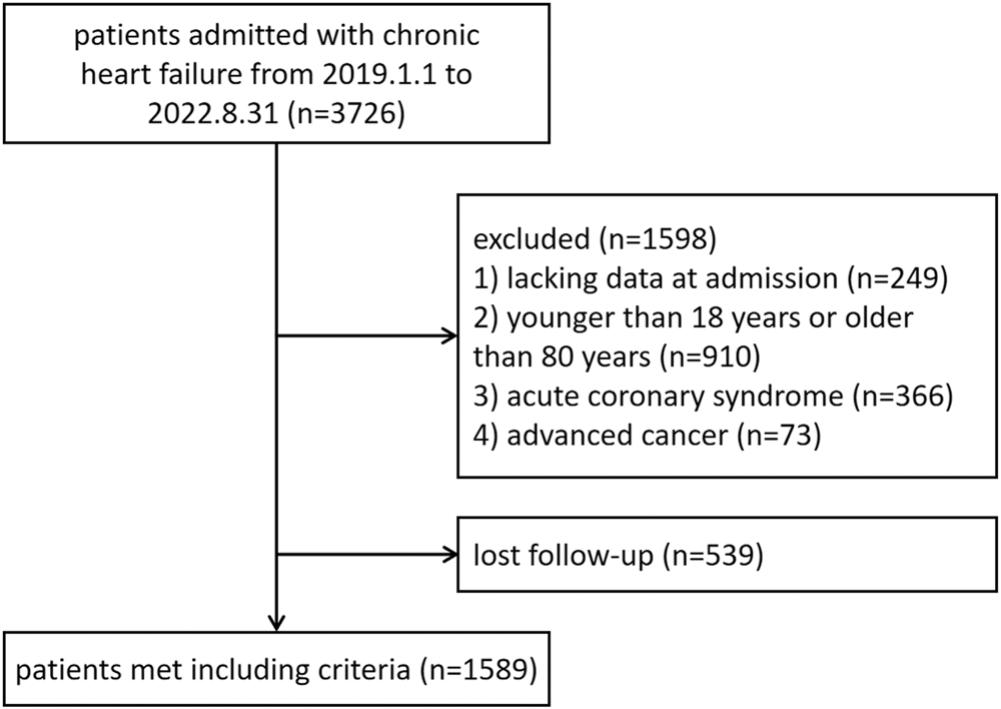
Flowchart of patient selection.

This retrospective analysis is conducted at a single‐center, Nanjing Drum Tower Hospital, consecutively encompassing 1589 hospitalized patients diagnosed with CHF from January 1, 2019 to August 31, 2022. The diagnosis of CHF adheres to the guidelines provided by the 2021 European Society for Cardiology Guidelines for the Diagnosis and Treatment of Acute and CHF [16]. In terms of exclusion criteria, we excluded patients who lacked clinical data at admission, patients with acute myocardial infarction, patients under the age of 18 or over the age of 80, patients with malignant tumors, and cases lost to follow‐up. Specific inclusion criteria is shown in Figure 1.

### 2.2 Data Collection and Definitions

Trained physicians extracted baseline demographic and clinical data from the electronic medical record system, including essential information such as age and gender, alongside body mass index (BMI), smoking history, alcohol consumption history, laboratory and instrumental examination results, comorbidities, and medication usage.

### 2.3 F‐SIRI Calculation

The formula for calculating SIRI is as follows: SIRI = neutrophil count (10^9^/L) × monocyte count (10^9^/L)/lymphocyte count (10^9^/L). The study population was randomly divided into training and test sets. 100 random splits were performed, with one‐third of participants in each split forming the test set. The mean Receiver Operating Characteristic (ROC) curve was constructed utilizing a threefold cross‐validation approach with 100 iterations. The optimal cut‐off values for SIRI and fibrinogen were determined to be 1.22 and 2.55 g/L, respectively. The F‐SIRI value was assigned as two for patients with both high fibrinogen (≥2.55 g/L) and high SIRI (≥1.22) levels. Patients with either a high fibrinogen or high SIRI level were assigned a value of 1. In addition, patients with both low fibrinogen (<2.55 g/L) and low SIRI (<1.22) levels were assigned a value of 0.

### 2.4 Endpoints and Follow Up

Following discharge, we performed regular patient follow‐ups every 6 months via telephone consultations and outpatient appointments. The median duration of follow‐up was 687 days. The endpoint events included all‐cause death, MAACEs and cardiovascular death. MACCEs were defined as a composite outcome consisting of non‐fatal stroke, non‐fatal myocardial infarction, exacerbation of HF requiring intravenous diuretic therapy or hospital readmission.

### 2.5 Statistical Analysis

Continuous variables are represented by mean ± standard deviation (SD) and median (interquartile range [IQR]), which are suitable for normally distributed and skewed variables, respectively. Categorical variables are expressed as numerical values (percentages) [17]. Patients were categorized into three groups based on their F‐SIRI levels. Analysis of variance (ANOVA) or Kruskal‐Wallis test was employed to compare continuous variables among these three groups. The *χ* ^2^ test was used to evaluate differences in categorical variables across the groups.

The Kaplan−Meier curve was utilized to calculate the cumulative event‐free survival rates for three endpoint events, while the log‐rank test was employed to compare the groups. Time to event was defined as the number of days from admission to either first event or censored date. Additionally, Multivariate Cox proportional hazards models were employed to examine the associations between F‐SIRI levels and the incidence rates of three major endpoint events. Before conducting these analyses, the validation of the proportional hazards assumption is performed by visualizing the Schoenfeld residuals. Baseline variables with a *p* value less than 0.05 or those demonstrating clear clinical significance are incorporated as potential confounding factors in the multivariate Cox proportional hazards models. Additionally, when evaluating multicollinearity within the multivariate regression model, a variance inflation factor below 10 is considered an acceptable threshold.

The final analysis comprised four multivariable regression models showed in Table 2. In all four models, we selected the lowest level of F‐SIRI as the reference standard. To capture the dose–response relationship between SIRI and the risk of three primary outcomes, we employed restricted cubic spline analysis with five nodes placed at the 5th, 27.5th, 50th, 72.5th, and 95th percentiles to ensure a comprehensive examination of the association.

Additionally, we conducted subgroup analysis to explore potential effect modifiers, including age (<70 vs. ≥70 years), gender (male vs. female), BMI (<24 vs. ≥24 kg/m^2^), history of diabetes mellitus (DM) (no vs. yes), history of atrial fibrillation (no vs. yes), history of previous myocardial infarction (no vs. yes) and history of chronic kidney disease (CKD) (no vs. yes). Likelihood ratio tests were utilized to assess the interaction effects among subgroups. We conducted continuous net reclassification improvement (NRI), integrated discrimination improvement (IDI) and C‐index analyses to assess the incremental predictive value of F‐SIRI beyond conventional risk factors.

In statistical analysis, the two‐sided significance level is established as *p* < 0.05. All statistical analyses were conducted using R software version 4.2.3 and SPSS for Windows version 26.

## 3 Results

### 3.1 Baseline Characteristics

The baseline characteristics of the 1589 enrolled patients categorized based on F‐SIRI levels are presented in Table 1. These patients had a median age of 66 years and a female representation of 32.9%. Individuals with hypertension, DM and CKD were significantly more prevalent in the highest F‐SIRI level. Additionally, elevated levels of BNP, FPG, HbA1c, PT, INR, fibrinogen, d‐dimer, neutrophil count, monocyte count, platelet, and FT4 were observed in highest level of F‐SIRI (all *p* < 0.05). Conversely, the highest F‐SIRI level exhibited lower sodium, eGFR, lymphocyte count, hemoglobin, TSH and FT3 levels (all *p* < 0.05). Regarding LVEF, three F‐SIRI level showed no statistical difference. We discovered that patients with higher F‐SIRI levels were less inclined to use ACEI/ARB/ARNI while being more likely to utilize antiplatelet agent, diuretics, statins and CCB (*p* < 0.05).

**Table 1:** Baseline clinical characteristics categorized by F‐SIRI group.

Characteristics	Overall (*n* = 1589)	F‐SIRI = 0 (*n* = 346)	F‐SIRI = 1 (*n* = 733)	F‐SIRI = 2 (*n* = 510)	*p* value
Female, *n* (%)	522 (32.85)	126 (36.42)	252 (34.38)	144 (28.24)	**0.0213**
Age (years)	66 [56, 72]	65 [54, 71]	66 [57, 72]	66 [57, 73]	0.0605
BMI (kg/m^2^)	24.70 [22.41, 27.47]	24.51 [22.38, 27.31]	24.80 [22.43, 27.55]	24.77 [22.41, 27.46]	0.8668
Heart rate (bpm)	78 [68, 90]	74 [66, 84]	77 [68, 89]	80 [69, 96]	**<0.0001**
SBP (mmHg)	129 [114, 145]	126 [111, 141]	129 [114, 143]	132 [117, 150]	**0.0002**
DBP (mmHg)	77 [68, 88]	79 [69, 88]	77 [68, 87]	78 [68, 91]	0.3344
Smoking, *n* (%)	474 (29.83)	85 (24.57)	227 (30.97)	162 (31.76)	0.0512
Alcohol, *n* (%)	279 (17.56)	49 (14.16)	132 (18.01)	98 (19.22)	0.1475
*Medical history, n (%)*					
Hypertension	924 (58.15)	174 (50.29)	424 (57.84)	326 (63.92)	**0.0004**
Hyperlipidemia	239 (15.04)	52 (15.03)	119 (16.23)	68 (13.33)	0.3714
Previous MI	205 (12.90)	40 (11.56)	86 (11.73)	79 (15.49)	0.1061
CKD	240 (15.10)	21 (6.07)	99 (13.51)	120 (23.53)	**<0.0001**
Diabetes mellitus	503 (31.66)	64 (18.50)	240 (32.74)	199 (39.02)	**<0.0001**
AF	595 (37.44)	148 (42.77)	266 (36.29)	181 (35.49)	0.0657
Pacemaker	182 (11.45)	47 (13.58)	81 (11.05)	54 (10.59)	0.3602
*Laboratory mesurement*					
BNP (pg/mL)	366 [147, 862]	267 [102, 658]	348 [141, 754]	496 [204, 1160]	**<0.0001**
TP (g/L)	64.4 [60.6, 68.7]	64.1 [60.4, 67.9]	65.2 [61.3, 69.3]	63.5 [59.8, 68.1]	**0.0004**
FPG (mmol/L)	5.0 [4.5, 5.9]	4.8 [4.4, 5.3]	5.0 [4.5, 5.8]	5.1 [4.5, 6.5]	**<0.0001**
HbA1c (%)	6.0 [5.6, 6.7]	5.8 [5.5, 6.3]	6.0 [5.6, 6.7]	6.2 [5.7, 7.2]	**<0.0001**
TG (mg/L)	1.1 [0.8, 1.6]	1.1 [0.8, 1.7]	1.2 [0.9, 1.6]	1.1 [0.8, 1.5]	**0.0017**
TC (mg/L)	3.8 [3.2, 4.6]	3.7 [3.2, 4.5]	3.9 [3.3, 4.7]	3.7 [3.0, 4.5]	**0.0248**
LDL‐C (mg/L)	2.1 [1.6, 2.8]	2.1 [1.6, 2.7]	2.2 [1.7, 2.8]	2.1 [1.5, 2.8]	**0.0316**
Calcium (mmol/L)	2.3 [2.2, 2.4]	2.3 [2.2, 2.4]	2.3 [2.2, 2.4]	2.3 [2.2, 2.4]	**0.0249**
eGFR (mL/min/1.73 m^2^)	89 [69, 108]	94.9 [79.7, 110.6]	91.9 [73.2, 109.3]	77.7 [52.9, 98.6]	**<0.0001**
PT (s)	11.8 [11.0, 12.9]	11.7 [11.0, 12.9]	11.6 [11.0, 12.6]	12.1 [11.3, 13.2]	**<0.0001**
INR	1.0 [1.0, 1.1]	1.0 [1.0, 1.1]	1.0 [1.0, 1.1]	1.1 [1.0, 1.2]	**<0.0001**
Fibrinogen (g/L)	2.9 [2.5, 3.5]	2.2 [2.0, 2.4]	3.0 [2.6, 3.4]	3.4 [3.0, 4.2]	**<0.0001**
D‐dimer (mg/L)	0.4 [0.2, 1.0]	0.3 [0.2, 0.7]	0.4 [0.2, 0.8]	0.6 [0.3, 1.4]	**<0.0001**
Neutrophil (10^9/L)	3.8 [3.0, 4.8]	3.0 [2.5, 3.6]	3.5 [2.8, 4.2]	5.1 [4.1, 6.3]	**<0.0001**
Lymphocyte (10^9/L)	1.6 [1.1, 2.0]	1.7 [1.3, 2.2]	1.6 [1.3, 2.0]	1.3 [1.0, 1.7]	**<0.0001**
Monocyte (10^9/L)	0.4 [0.3, 0.6]	0.4 [0.3, 0.4]	0.4 [0.3, 0.5]	0.6 [0.5, 0.7]	**<0.0001**
Hemoglobin (g/L)	134 [120, 147]	137 [124, 148]	134 [121, 148]	131 [114, 144]	**0.0001**
Platelet (10^9/L)	177 [141, 217]	157 [122, 189]	182 [146, 219]	188 [146, 233]	**<0.0001**
TSH (mIU/L)	2.3 [1.5, 3.5]	2.4 [1.5, 3.8]	2.3 [1.5, 3.3]	2.2 [1.3, 3.6]	0.0921
FT3 (IU/mL)	4.3 [3.8, 4.8]	4.5 [4.1, 5.0]	4.4 [3.9, 4.9]	4.1 [3.5, 4.6]	**<0.0001**
FT4 (IU/mL)	17.5 [15.4, 19.6]	17.3 [15.1, 19.3]	17.4 [15.5, 19.2]	17.7 [15.4, 20.3]	**0.02**
*Echocardiography*					
LVEF (%)	40 [32, 52]	41 [33, 52]	40 [31, 52]	40 [31, 50]	0.3596
IVSTd (cm)	0.9 [0.8, 1.0]	0.9 [0.8, 1.0]	0.9 [0.8, 1.0]	0.9 [0.8, 1.0]	**0.0027**
LVPWTd (cm)	0.9 [0.8, 1.0]	0.9 [0.8, 1.0]	0.9 [0.8, 1.0]	0.9 [0.8, 1.0]	**0.0102**
LVDd (cm)	5.9 [5.3, 6.6]	5.9 [5.3, 6.7]	5.9 [5.3, 6.6]	5.8 [5.3, 6.6]	0.9991
AoD (cm)	3.2 [3.0, 3.4]	3.2 [3.0, 3.4]	3.2 [3.0, 3.5]	3.2 [3.0, 3.4]	0.5393
LAD (cm)	4.7 [4.3, 5.2]	4.6 [4.2, 5.2]	4.7 [4.3, 5.2]	4.7 [4.3, 5.2]	0.922
*Medications at discharge, n (%)*					
ACEI/ARB/ARNI	1121 (70.55)	265 (76.59)	518 (70.67)	338 (66.27)	**0.0051**
β‐blocker	1238 (77.91)	274 (79.19)	569 (77.63)	395 (77.45)	0.8079
MRA	1022 (64.32)	222 (64.16)	484 (66.03)	316 (61.96)	0.3371
SGLT2i	307 (19.32)	66 (19.08)	155 (21.15)	86 (16.86)	0.1689
Antiplatelet agent	645 (40.59)	108 (31.21)	302 (41.20)	235 (46.08)	**0.0001**
Digoxin	188 (11.83)	43 (12.43)	82 (11.19)	63 (12.35)	0.7628
Statins	960 (60.42)	183 (52.89)	458 (62.48)	319 (62.55)	**0.0053**
CCB	245 (15.42)	32 (9.25)	113 (15.42)	100 (19.61)	**0.0002**

Abbreviations: ACEI, angiotensin‐converting enzyme inhibitors; AoD, aortic diameter; ARB, angiotensin receptor blockers; ARNI, angiotensin receptor II blocker‐neprilysin inhibitor; BMI, body mass index; BNP, B‐type natriuretic peptide; CCB, calcium channel blockers; eGFR, estimated glomerular filtration rate; FPG, fasting plasma glucose; FT3, free triiodothyronine; FT4, free thyroxine; INR, international normalized ratio; IVSTD, interventricular septal thickness; LAD, left atrial diameter; LDL‐C, low‐density lipoprotein C; LVEF, left ventricular ejection fraction; LVDd, left ventricular diastolic diameter; LVPWT, left ventricular posterior wall thickness; MRA, mineralocorticoid receptor antagonist; PT, prothrombin time; TC, total cholesterol; TG, triglyceride; TP, total protein; TSH, thyroid stimulating hormone; UA, uric acid;

### 3.2 SIRI as a Continuous Variable

In Supporting Information S1: Figure 1A and Supporting Information S1: Figure 1B, the restricted cubic spline models demonstrated a rapid initial increase in the risk of both outcomes, followed by a plateau or even a decrease. The risk of all‐cause death exhibited a significant increase when SIRI exceeded 1.01. Moreover, for SIRI values above 1.01, there was also an observed rise in the hazard ratio (HR) associated with MACCEs.

### 3.3 Predictive Ability of the F‐SIRI Level for Three Primary Outcomes

During the follow‐up process, patients with all‐cause death included 24 (6.94%) in F‐SIRI = 0 group, 82 (11.19%) in F‐SIRI = 1 group and 101 (19.80%) in F‐SIRI = 2 group. Individuals with MACCEs included 82 (23.7%) in F‐SIRI = 0 group, 197 (26.88%) in F‐SIRI = 1 group and 183 (35.88%) in F‐SIRI = 2 group. Individuals with cardiovascular death included 24 (6.94%) in F‐SIRI = 0 group, 82 (11.19%) in F‐SIRI = 1 group and 101 (19.80%) in F‐SIRI = 2 group.

**Figure 2:**
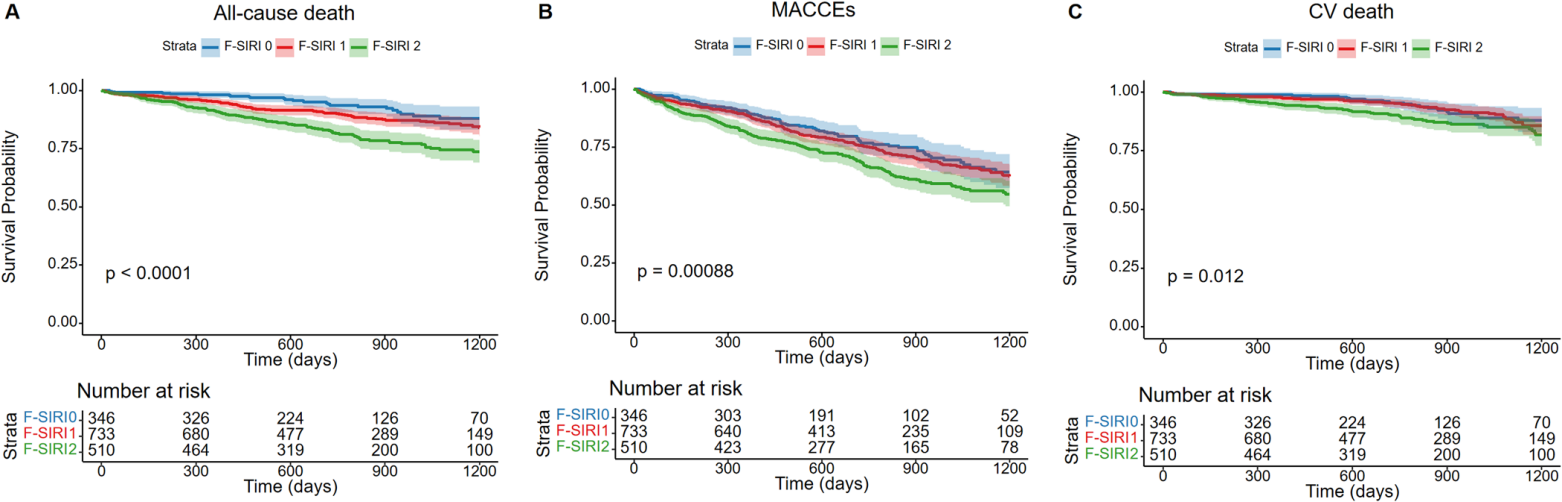
Kaplan−Meier analysis of (A) All‐cause death, (B) MACCEs and (C) CV death in various F‐SIRI groups. CV death, cardiovascular death; F‐SIRI, fibrinogen and systematic inflammation response index; MACCEs, major adverse cardiac and cerebral events.

**Figure 3:**
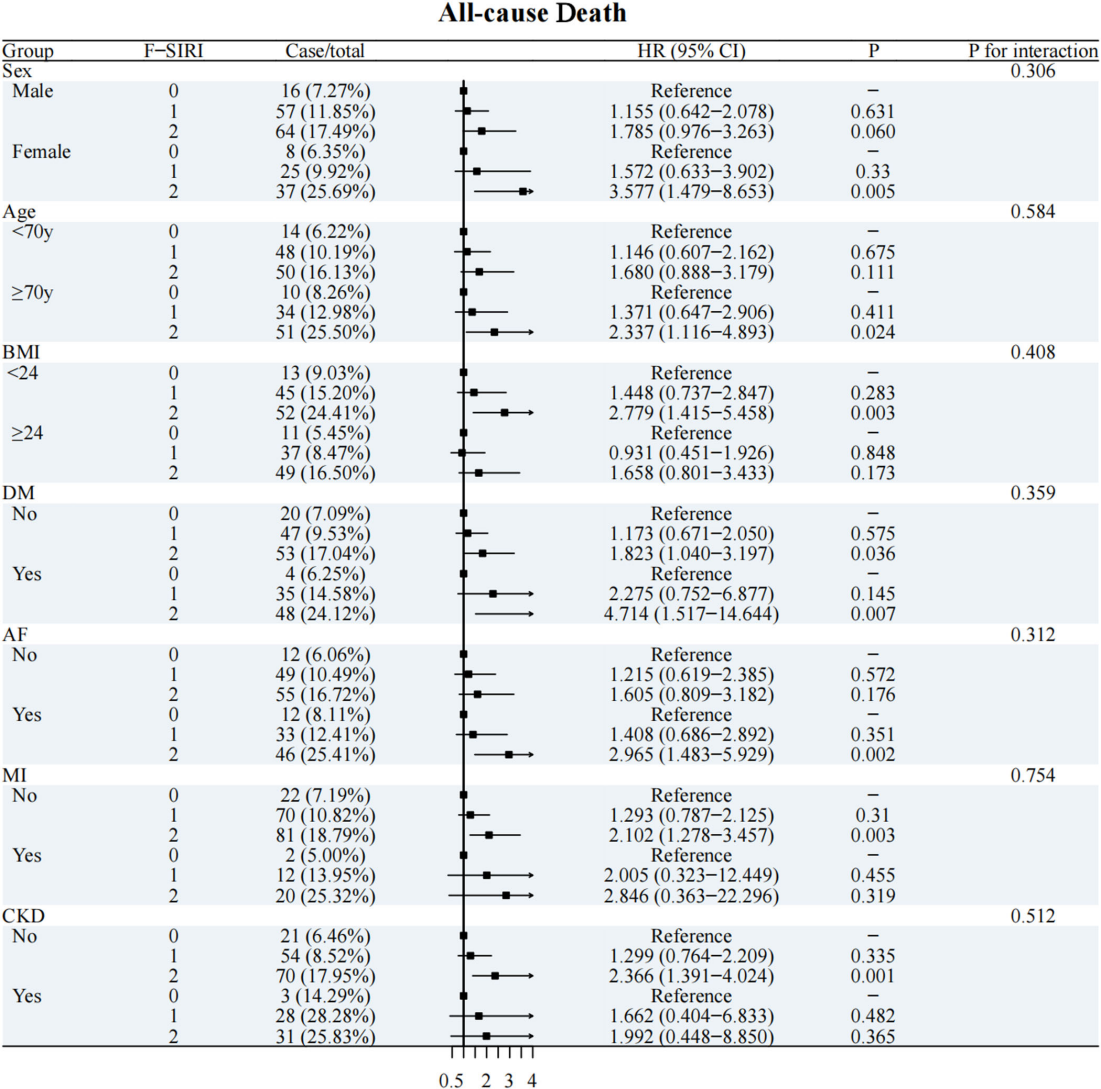
Forest plot of All‐cause death according to different subgroups.

The event‐free survival rate of three primary outcomes, as indicated by the SIRI and fibrinogen cut‐off values using Kaplan–Meier curves, is depicted in Supporting Information S1: Figures 2A–C, 3A–C. Patients with higher SIRI levels exhibited a significant increase in the cumulative incidence rates of three primary outcomes (Supporting Information S1: Figure 2A–C) (*p* < 0.05, log‐rank test). Furthermore, group with elevated fibrinogen levels demonstrated a significantly higher risk of all‐cause death compared to the group with lower fibrinogen levels (Supporting Information S1: Figure 3A) (*p* < 0.05, log‐rank test). However, there was no observed significant difference between the high and low fibrinogen level groups in terms of cumulative incidence rates of MACCEs and cardiovascular death (Supporting Information S1: Figure 3B,C) (*p* > 0.05, log‐rank test).

The Kaplan–Meier curves demonstrated significant differences among three F‐SIRI levels in three predefined primary endpoints (Figure 2A–C) (*p* < 0.05, log‐rank test). Table 2 showed that F‐SIRI = 2 is significantly associated with an increased incidence rate of all‐cause death (Model 4, adjusted HR of F‐SIRI = 2: 2.37, 95% CI: 1.46–3.83, *p* < 0.001), while no marked differences were observed between the F‐SIRI = 1 and F‐SIRI = 0. However, the association between F‐SIRI and the risk of MACCEs as well as cardiovascular death was no longer statistically significant after adjusting for multiple confounding factors.

**Table 2:** HR (95% CI) of primary outcomes based on F‐SIRI in the four Models.

			Model 1		Model 2		Model 3		Model 4	
Outcomes	F‐SIRI groups	Events, *n* (%)	HR (95% CI)	*p* value	HR (95% CI)	*p* value	HR (95% CI)	*p* value	HR (95% CI)	*p* value
All‐cause death	0	24 (6.94)	Ref.		Ref.		Ref.		Ref.	
	1	82 (11.19)	1.63 (1.03−2.57)	0.036	1.63 (1.01−2.61)	0.044	1.60 (0.99−2.57)	0.053	1.49 (0.92−2.42)	0.102
	2	101 (19.80)	3.00 (1.91−4.72)	< 0.001	2.52 (1.57−4.06)	< 0.001	2.49 (1.54−4.01)	< 0.001	2.37 (1.46−3.83)	< 0.001
MACCEs	0	82 (23.70)	Ref.		Ref.		Ref.		Ref.	
	1	197 (26.88)	1.10 (0.85−1.43)	0.462	1.12 (0.86−1.46)	0.398	1.10 (0.84−1.43)	0.492	1.09 (0.84−1.43)	0.506
	2	183 (35.88)	1.52 (1.17−1.99)	0.002	1.32 (1.00−1.74)	0.051	1.30 (0.99−1.72)	0.061	1.30 (1.0.98−1.72)	0.064
CV death	0	24 (6.94)	Ref.		Ref.		Ref.		Ref.	
	1	82 (11.19)	1.19 (0.72−1.95)	0.501	1.18 (0.70−1.96)	0.536	1.23 (0.73−2.05)	0.053	1.18 (0.70−1.98)	0.538
	2	101 (19.80)	1.94 (1.18−3.20)	0.009	1.65 (0.98−2.78)	0.059	1.73 (1.03−2.91)	0.040	1.63 (0.97−2.76)	0.067

*Note:* Model 1: adjusted for gender, age, BMI, SBP, DBP and heart rate. Model 2: adjusted for Model 1+Left atrial diameter, LVEF, BNP, total protein, TG, TC, LDL‐C, calcium, eGFR, PT, INR, TSH, FT3, hemoglobin and HbA1c. Model 3: adjusted for Model 2+history of previous myocardial infarction, diabetes mellitus, hyperlipidemia, hypertension, atrial fibrillation, chronic kidney disease and pacemaker implantation. Model 4: adjusted for Model 3+ use of ACEI/ARB/ARNI, β‐blocker, MRA, SGLT2i, statins, digoxin, and CCB.

Abbreviations: CI, confidence interval; CV death, cardiovascular death; F‐SIRI, Fibrinogen and systematic inflammatory response index; HR, hazard ratio; MACCEs, the major adverse cardiac and cerebral events.

### 3.4 Predictive Efficacy of F‐SIRI on Three Primary Endpoints in CHF Patients

Supporting Information S1: Table 1 showed the incremental predictive ability of SIRI, Fib, and F‐SIRI based on this multivariate model. F‐SIRI slightly improved the prediction of MACCEs in multivariate models (change in NRI, *p* < 0.0001; IDI, *p* < 0.01), whereas adding SIRI or Fib individually did not show notable improvements. The incremental prognostic value of F‐SIRI was similar to that of SIRI or Fib in predicting all‐cause death.

### 3.5 Subgroup Analysis

The subgroup analysis revealed consistent associations between the three F‐SIRI levels and the risk of all‐case‐death (Figure 3), MACCEs (Supporting Information S1: Figure 4) and cardiovascular death (Supporting Information S1: Figure 5) among different subgroups, including age, sex, BMI, history of diabetes mellitus, history of previous myocardial infarction and chronic kidney disease. No significant interactions were found between the F‐SIRI level and any variables in subgroup analyses (all *p* values for interaction > 0.05).

## 4 Discussion

In this retrospective study, the relationship between F‐SIRI and the risk of all‐cause death, MACCEs and cardiovascular death in CHF patients was explored in a real clinical setting. This study firstly propose that the combination of fibrinogen and SIRI may contribute to stratifying the risk of adverse outcomes in CHF patients. In our cohort study, we found that higher levels of F‐SIRI were associated with an increased risk of all‐cause death in CHF patients, and had no significant or weak association with an increased risk of MACCEs and cardiovascular death. Moreover, the addition of F‐SIRI increased the predictive power of multivariate models based on traditional risk factors for all‐cause death and MACCEs. Additionally, this correlation was consistent across different subgroups such as age, gender, BMI, history of DM, previous MI, and CKD. The multivariable Cox regression model adjusted for multiple factors showed that F‐SIRI was a predictor for all‐cause death in CHF patients independent of other traditional factors.

### 4.1 Association Between Systemic Inflammation and Cardiovascular Disease

In recent years, inflammation has emerged as a potential mechanism contributing to the development of HF, with bidirectional interactions between inflammation and HF [6, 18, 19, 20]. Researchers have observed that increased levels of pro‐inflammatory cytokines in serum are associated with unfavorable outcomes in patients with CHF, both those with reduced ejection fraction (HFrEF) and preserved ejection fraction (HFpEF). However, these associations appear to be less pronounced compared to patients with autoimmune diseases or acute infections, suggesting that chronic low‐grade inflammation may play a significant role in perpetuating adverse clinical outcomes in individuals with CHF [21, 22, 23, 24]. Furthermore, several studies propose that inflammation serves as a crucial pathophysiological characteristic of CHF and inflammatory markers can aid in the diagnosis and prognosis evaluation of CHF patients [25, 26, 27].

SIRI, serving as a novel composite inflammatory biomarker, not only boasts the advantages of cost‐effectiveness, easy accessibility and reproducible detection but also exhibits a close correlation with cardiovascular diseases. In a retrospective study involving elderly patients with HF, SIRI demonstrated significant correlations with all‐cause mortality as well as length of hospitalization or ICU stay [11]. Another investigation highlighted the efficacy of SIRI in predicting long‐term mortality following valve surgery [28]. Furthermore, elevated levels of SIRI were found to be linked to increased risks of both all‐cause and cardiovascular mortality among individuals diagnosed with hypertension [29]. Therefore, we can infer that SIRI may have great potential in predicting CHF across a broader age span.

The underlying pathophysiological mechanism responsible for the association between SIRI and poor prognosis in patients with CHF remains elusive. Numerous studies have consistently demonstrated that inflammation cells and the release of inflammatory factors play crucial roles in various key aspects of HF, including myocardial injury, heart repair, and reconstruction [20, 26, 30]. HF is often accompanied by cardiomyocytes injury. Damaged cardiomyocytes can cause the activation and infiltration of neutrophils. Neutrophils secretes reactive oxygen species and proteases to aggravate endothelial cell injury, and secrete inflammatory factors to recruit monocytes and induce the activation of macrophages, which further aggravate the inflammatory response [30, 31, 32]. Altered cardiac function can elicit a feedback mechanism that triggers activation of the neuroendocrine system, ultimately leading to diminished survival of lymphocytes [33, 34, 35].

### 4.2 Association Between Fibrinogen and Cardiovascular Disease

Fibrinogen, a liver‐synthesized acute‐phase protein, is the predominant coagulation factor in plasma [36]. It has been proposed that fibrinogen serves as an independent predictor for myocardial infarction [37]. The level of fibrinogen exerts an impact on primary endpoint events among patients with HF. This could be attributed to the mechanism wherein tissue ischemia and hypoxia during HF result in vascular endothelial cell damage, triggering activation of the endogenous coagulation pathway. Consequently, this leads to a hypercoagulable state and microthrombosis, further exacerbating HF [38].

### 4.3 Rationality of Combining Fibrinogen and SIRI

Fibrinogen combined with SIRI, or F‐SIRI, serves as a readily accessible and straightforward indicator of inflammation and coagulation status, thereby potentially enhancing risk stratification in patients with CHF. This study represents the first attempt to integrate fibrinogen and SIRI into a prognostic model for CHF patients, demonstrating that this combination improves the predictive accuracy for adverse outcomes in CHF patients.

Precise regulation of the inflammatory response has emerged as a novel strategy for intervening in CHF [6, 24, 27]. Incorporating F‐SIRI as an additional assessment indicator within the risk evaluation and disease prognosis system for CHF will enhance clinical capabilities to identify and manage high‐risk cohorts susceptible to cardiovascular events. Nevertheless, it is important to acknowledge that F‐SIRI represents a non‐specific inflammatory marker and does not serve as a definitive prognostic indicator for CHF. Therefore, further investigation into key molecules and associated mechanisms involved in diverse inflammatory pathways is imperative to discover superior inflammatory biomarkers.

Consequently, F‐SIRI possesses substantial potential for predicting the prognosis of CHF patients while providing valuable clinical guidance for CHF management.

### 4.4 Limitations

There are several limitations that need to be considered in this study. Firstly, we cannot determine the association between F‐SIRI and longer outcomes in CHF patients due to limited observation time and sample size. Secondly, F‐SIRI was calculated only once from the baseline data at admission, which may not accurately reflect the changes in systemic inflammatory state over time. Furthermore, data bias cannot be fully avoided, although considerable efforts had been made to reduce the impact of confounding factors. These findings need to be validated in a prospective cohort study.

## 5 Conclusion

This study found association between elevated F‐SIRI levels and poor prognosis in CHF patients. An elevated level of F‐SIRI could function as a predictor for all‐cause death, but its ability to predict MACCEs and cardiovascular death is limited. In conclusion, F‐SIRI should be taken into consideration when managing CHF patients.

## Author Contributions

**Rong Gu, Biao Xu and Xue Bao:** conception and design of this study. **Xiaoli Liu:** writing original draft. **Xiaoli Liu, Heyu Chu and Guo Song:** data collection and management. **Xiaoli Liu and Xue Bao:** statistical analysis. **Yi Wang, Junfeng Duan and Xi Tan:** patient follow‐up. **Rong Gu and Xue Bao:** manuscript revision and data review. All authors have read and approved the manuscript.

## Ethics Statement

This study adhered to the principles of the Declaration of Helsinki and obtained approval from the Ethics Committee of Nanjing Drum Tower Hospital (2023‐428‐01).

Due to the retrospective design of the study, the institutional review board deemed it appropriate to waive the necessity for informed consent and rigorously upheld patient confidentiality.

## Conflicts of Interest

The authors declare no conflicts of interest.

## Supporting information

Supplementary Information(R2).

## Data Availability

The data that support the findings of this study are available on request from the corresponding author. The data are not publicly available due to privacy or ethical restrictions.
