# Supplementary material for: Association Between F‐SIRI and Adverse Prognosis in Patients With Chronic Heart Failure
Source: Clin Cardiol. 2025 Jun 16;48(6):e70166. doi: 10.1002/clc.70166 (PMC12168489; doi:10.1002/clc.70166)
Supplement: Supplementary file 1 — Supplementary Information(R2). [file CLC-48-e70166-s001.docx]

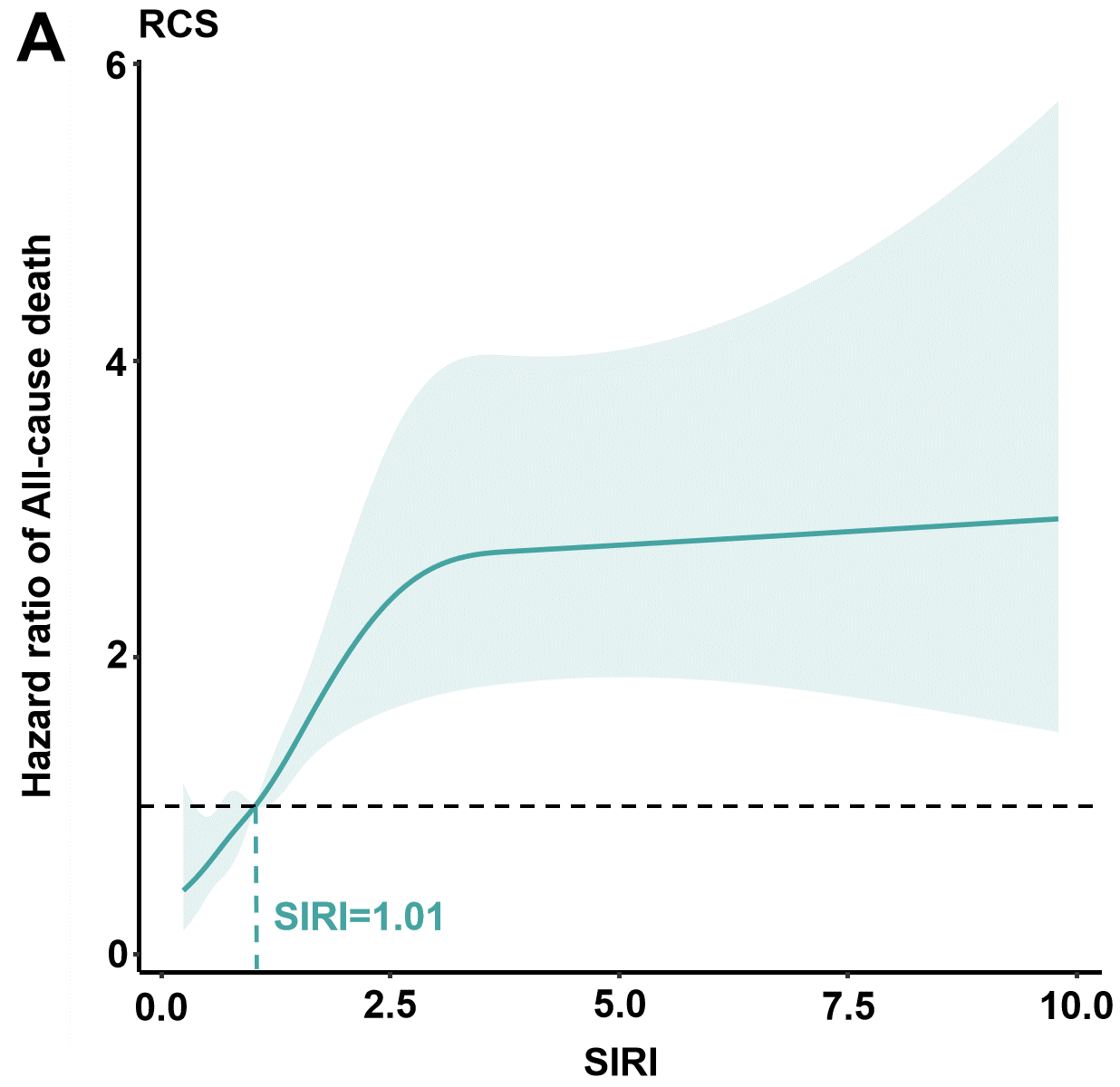

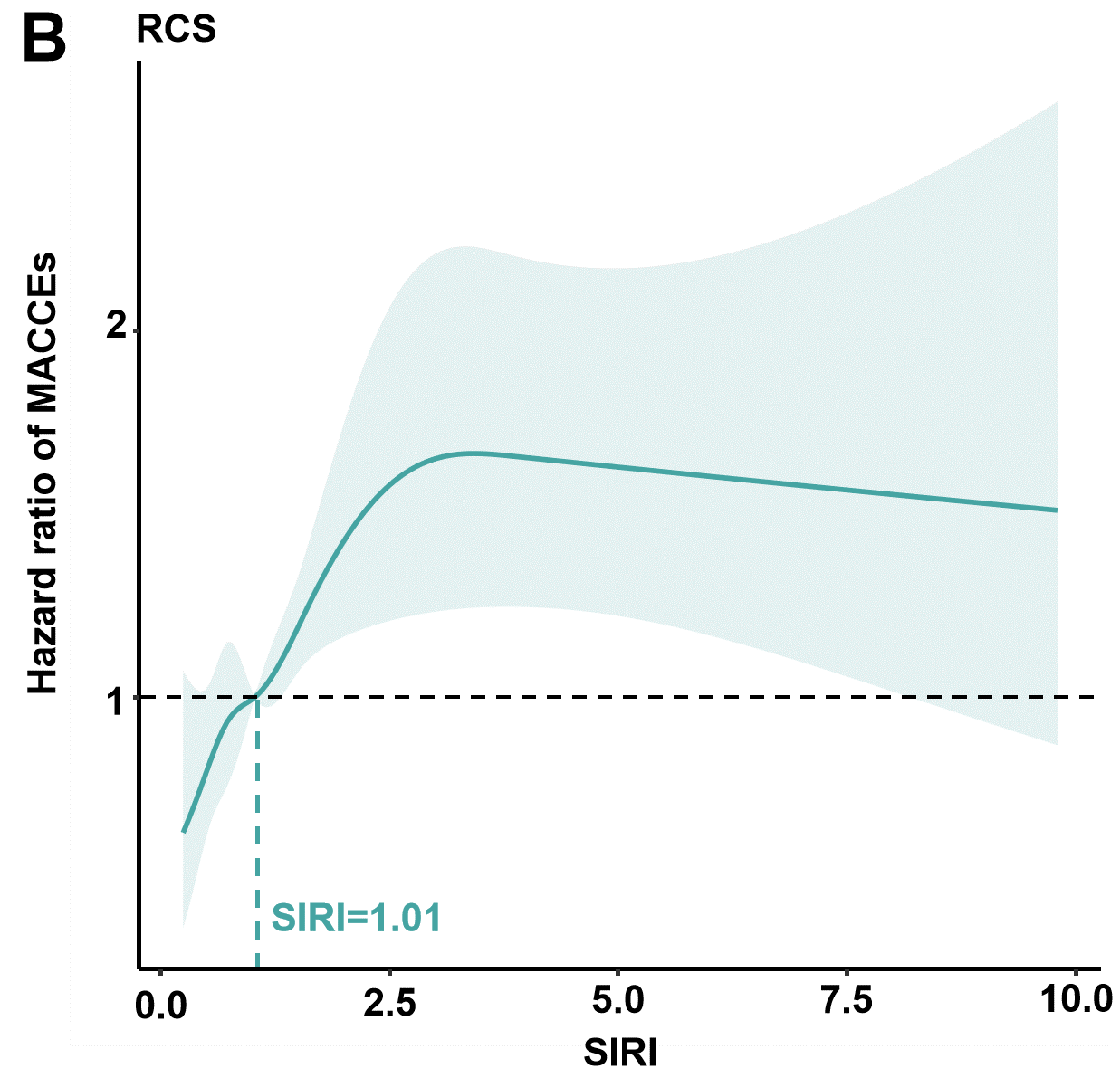


**Fig.S1** Hazard ratios for (**A**) All-cause death and (**B**) MACCEs based on RCS for SIRI.

SIRI, systematic inflammation response index. RCS, restricted cubic spines.


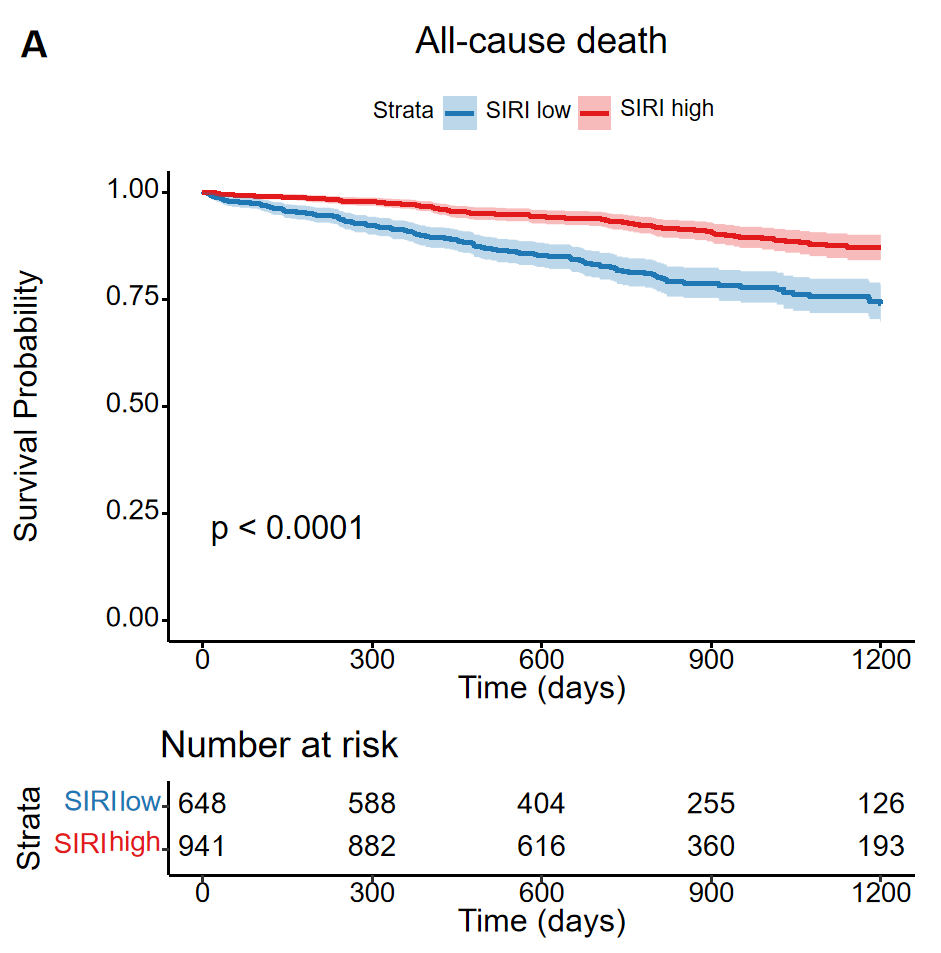

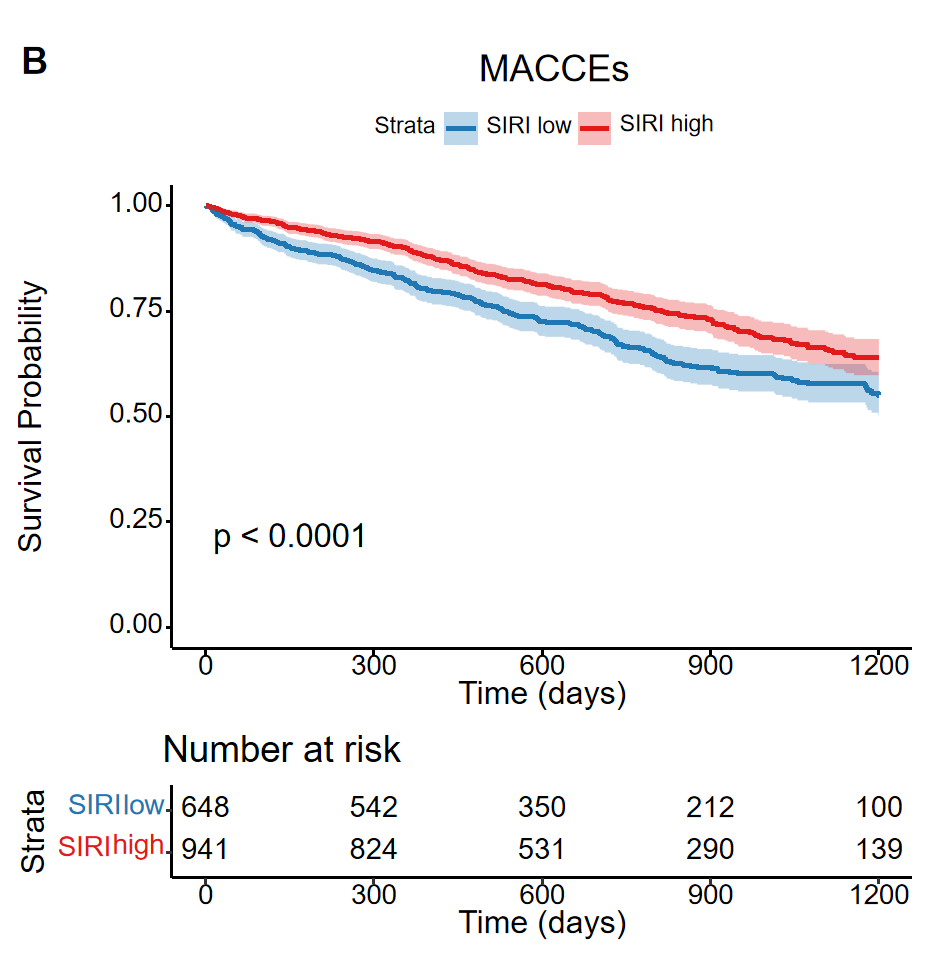

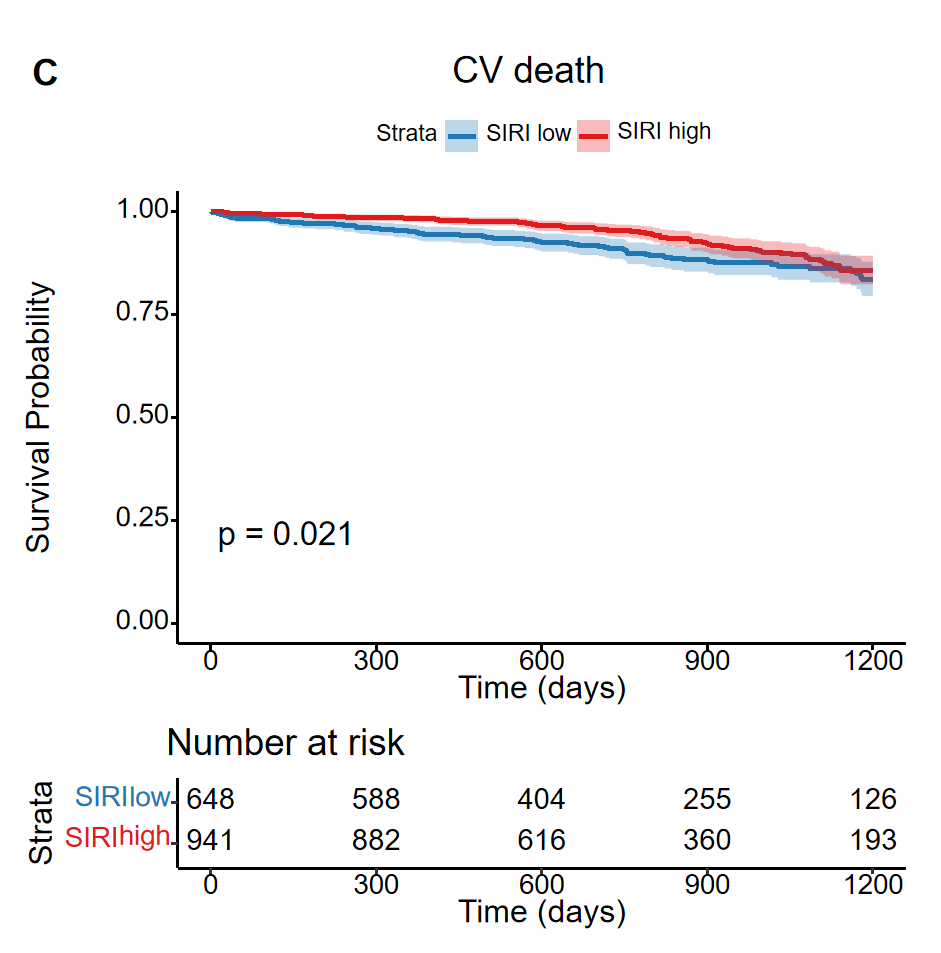


**Fig.S2** Kaplan-Meier analysis of (**A**) All-cause death, (**B**) MACCEs and (**C**) CV death in various SIRI groups.

SIRI, systematic inflammation response index. MACCEs, major adverse cardiac and cerebral events. CV death, cardiovascular death.


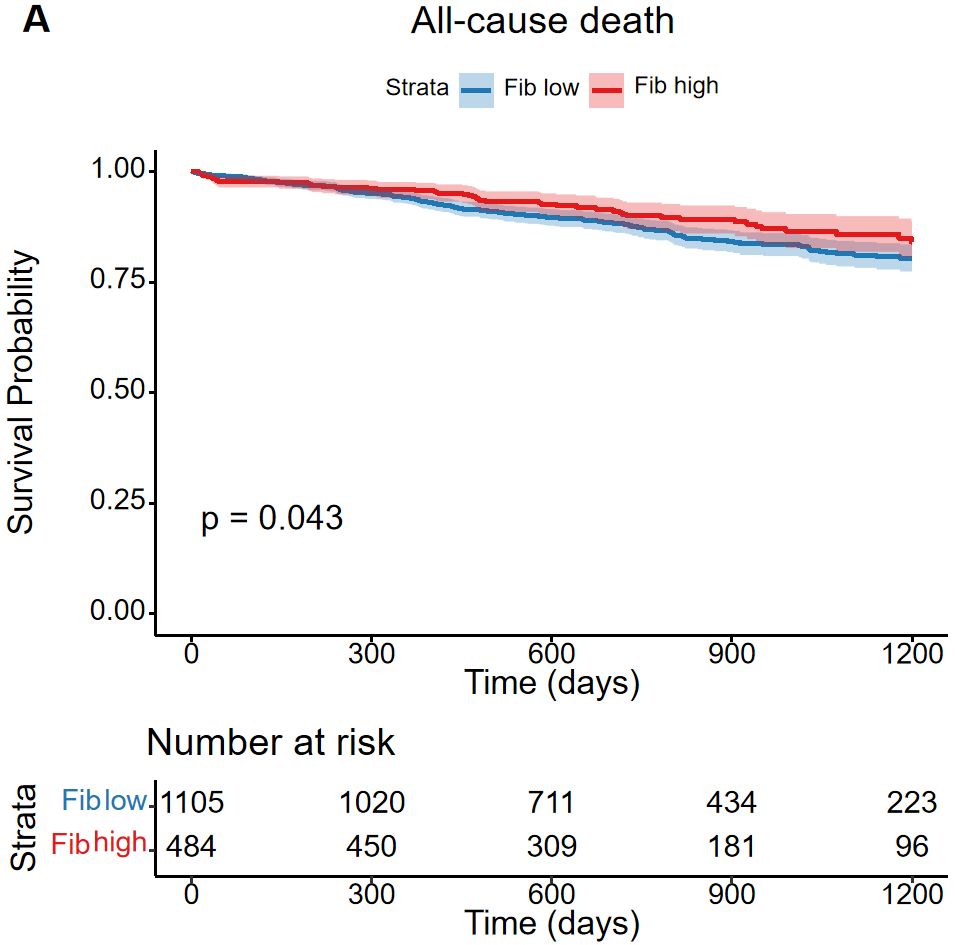

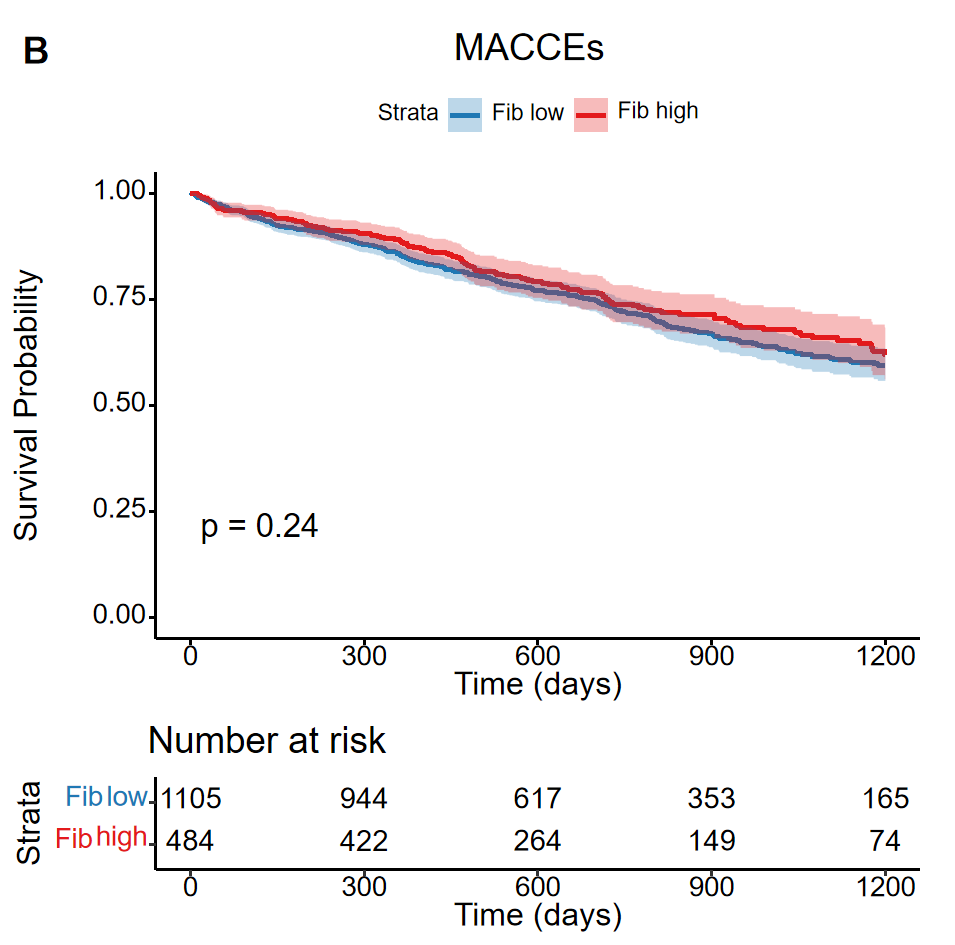


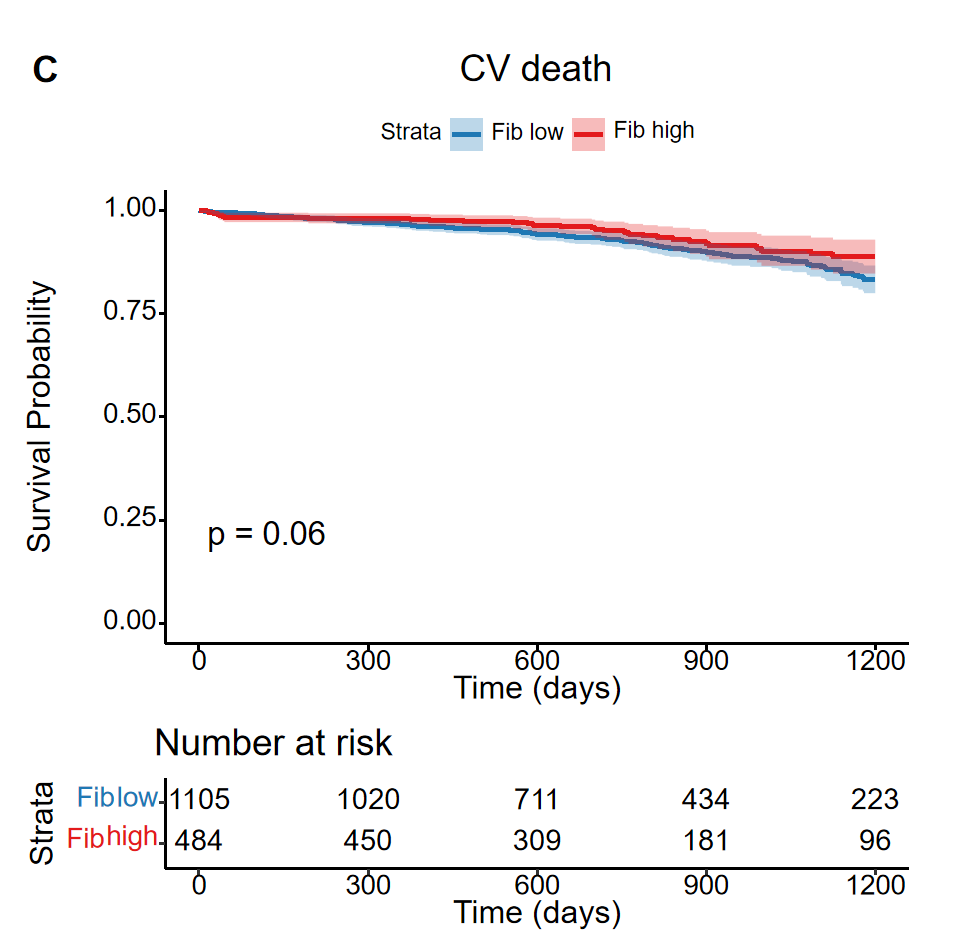


**Fig.S3** Kaplan-Meier analysis of (**A**) All-cause death, (**B**) MACCEs and (**C**) CV death in various fibrinogen groups.

MACCEs, major adverse cardiac and cerebral events. Fib, fibrinogen. CV death, cardiovascular death.

**Table S1.** Performance Metrics of SIRI, Fib and F-SIRI for all-cause death, MACCEs and CV death#.

| **Outcomes** |  | **NRI** | **IDI** | **C-index** |
| --- | --- | --- | --- | --- |
| All-cause death | Multivariate model † | Ref. | Ref. | Ref. |
|  | Multivariate model+SIRI | 0.0162 | 0.0147** | 0.7615 |
|  | Multivariate model+Fib | 0.0147 | 0.0103** | 0.7571 |
|  | Multivariate model+F-SIRI | 0.0104 | 0.0158** | 0.7650 |
| MACCEs | Multivariate model † | Ref. | Ref. | Ref. |
|  | Multivariate model+SIRI | 0.0024 | 0.0019 | 0.6616 |
|  | Multivariate model+Fib | 0.0117 | 0.0004 | 0.6601 |
|  | Multivariate model+F-SIRI | 0.0535**** | 0.0042** | 0.6630 |
| CV death | Multivariate model † | Ref. | Ref. | Ref. |
|  | Multivariate model+SIRI | 0.0225 | 0.0006 | 0.7293 |
|  | Multivariate model+Fib | 0.0005 | 0.0062* | 0.7346 |
|  | Multivariate model+F-SIRI | 0.0173 | 0.0032 | 0.7333 |

SIRI, systematic inflammatory response index; Fib, fibrinogen; F-SIRI, fibrinogen and systematic inflammatory response index; MACCEs, the major adverse cardiac and cerebral events; CV death, cardiovascular death; NRI, Net Reclassiﬁcation Improvement; IDI, Integrated Discrimination Improvement.

# Comparisons are made between multivariate model with and without SIRI, Fib or F-SIRI.

† Multivariate model includes age, sex, systolic blood pressure, left ventricular ejection fraction, B-type natriuretic peptide, HbA1c, low-density lipoprotein C, history of myocardial infarction, history of atrial fibrillation, implantation of pacemaker, angiotensin-converting enzyme inhibitors/angiotensin receptor blockers/angiotensin receptor II blocker-neprilysin inhibitor, β-blocker, mineralocorticoid receptor antagonist, sodium-glucose co-transporter 2 inhibitor.

**p*＜0.05; ** *p*＜0.01; ****p*＜0.001; **** *p*＜0.0001.


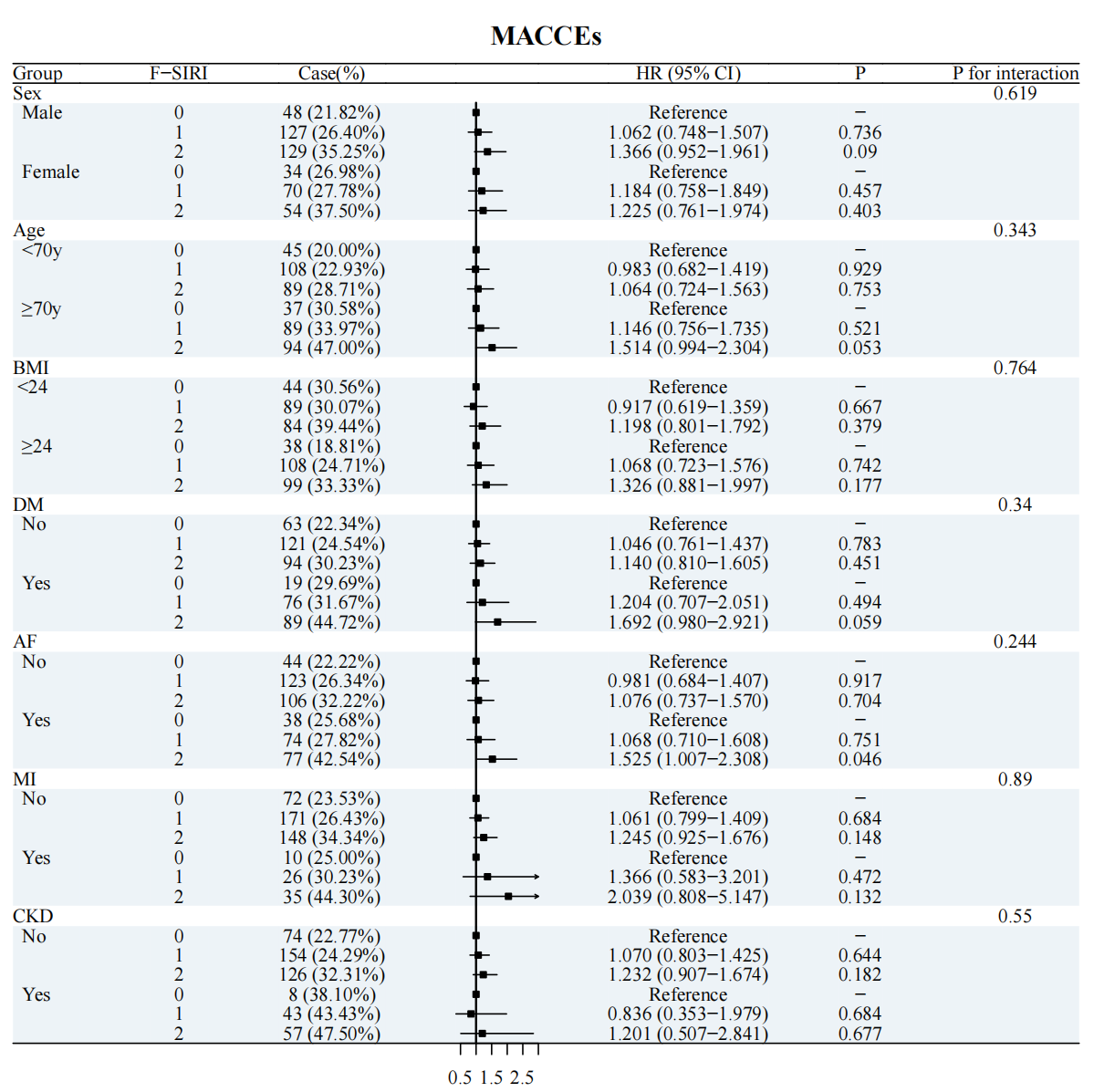


**Fig.S4** Forest plot of MACCEs according to different subgroups.


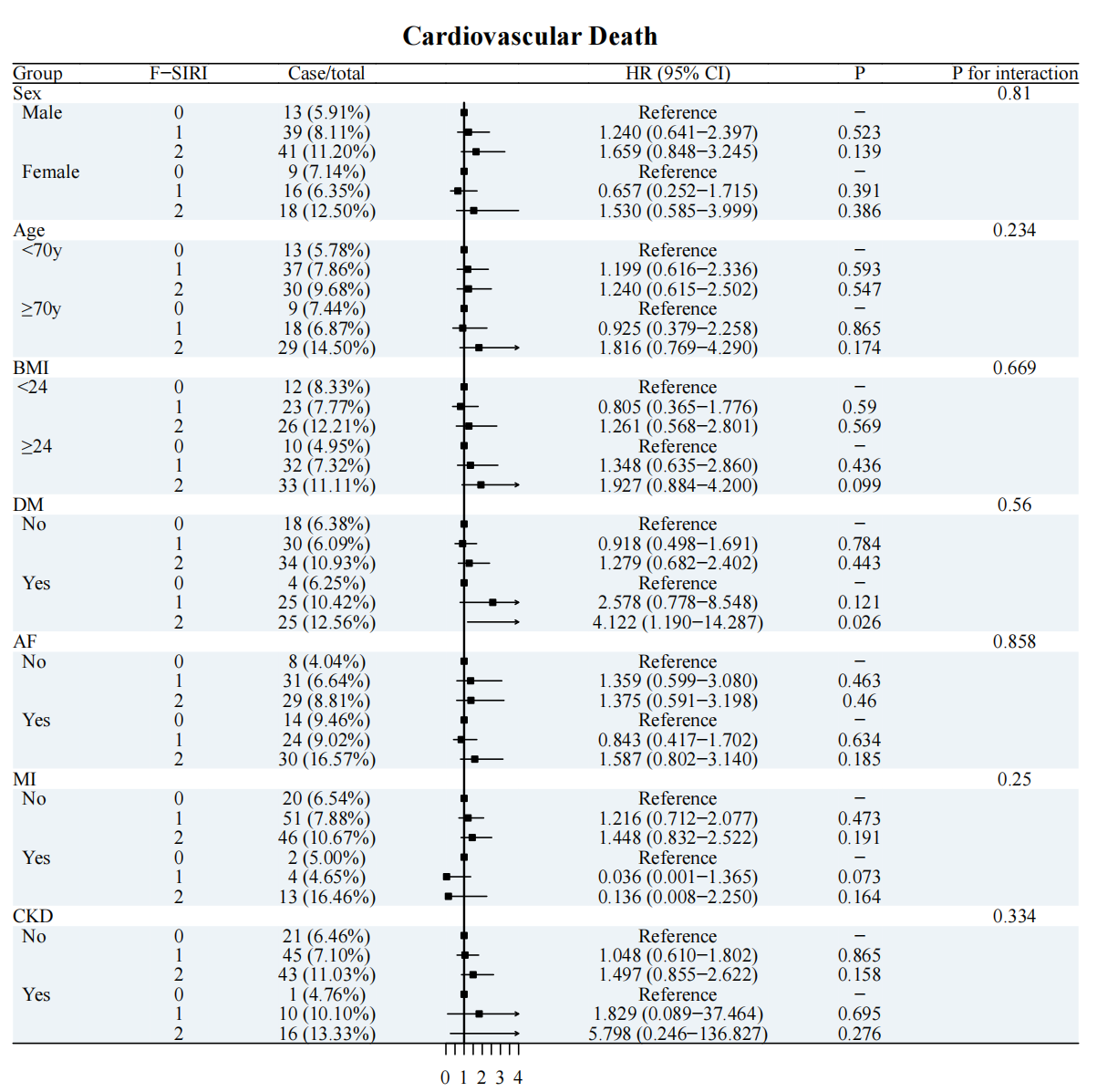


**Fig.S5** Forest plot of Cardiovascular death according to different subgroups.
